# Completion lobectomy for unanticipated pN1 disease on postoperative pathology after segmentectomy for cT1N0 lung cancer: Prevention of pleural adhesion by using fibrin glue

**DOI:** 10.1186/1749-8090-10-S1-A245

**Published:** 2015-12-16

**Authors:** Yoshihiro Miyata, Tsuyoshi Mimura, Yasuhiro Tsutani, Masahiro Ito, Keizo Misumi, Norifumi Tsubokawa, Yuichiro Kai, Atsusshi Kagimoto, Jun Hihara, Morihito Okada

**Affiliations:** 1 Department of Surgical Oncology, Hiroshima University, Hiroshima, Japan

## Background/Introduction

Segmentectomy is an anatomic sublobar resection that has recently been introduced for small lesions in cases of lung cancer.

## Aims/Objectives

In completion lobectomy after segmentectomy, pleural adhesion in the hilar vessels and inter-segmental plane makes the procedure most difficult. Preventing adhesion could reduce intraoperative bleeding and operation time in cases of reoperation.

## Method

Here, we present a case of right S9+10 segmentectomy followed by completion lobectomy for unanticipated nodal metastasis of cT1N0/pN1 lung cancer, in which pleural adhesion was prevented by coating with fibrin glue.

## Results

We describe the case of a 54-year-old man who underwent completion lobectomy for unanticipated hilar nodal metastasis reported on postoperative pathologic examination 1 month after right S8+9 segmentectomy for clinical T1N0 lung cancer. In the initial surgery, we used electrocautery without a stapler to divide the inter-segmental plane. The entire dissected inter-segmental plane was covered with absorbable mesh and fibrin glue. At re-thoracotomy, pleural adhesion at the inter-segmental plane was never observed although there were adhesions at parietal pleura not covered with fibrin glue.

## Discussion/Conclusion

Covering the inter-segmental plane with fibrin glue may be useful not only for preventing air leakage but also for preventing pleural adhesion.

## Consent

Written informed consent was obtained from the patient for publication of this abstract and any accompanying images. A copy of the written consent is available for review by the Editor of this journal.

